# Outcomes of neutropenic patients with severe sepsis on a specialist cancer ICU

**DOI:** 10.1186/cc13435

**Published:** 2014-03-17

**Authors:** S Jhanji, S Hallam, T Wigmore

**Affiliations:** 1 Royal Marsden NHS Trust, London, UK

## Introduction

The hospital survival rate for patients with septic shock remains at approximately 50% [1]. Critically ill cancer patients have often been considered poor candidates for ICU management due to the perception of poor outcomes for this group, in particular in the presence of neutropenia. There is a paucity of data supporting this. The objective of this study was to describe clinical outcomes for a group of septic neutropenic patients admitted to a cancer ICU.

## Methods

All neutropenic patients (neutrophils <1,000/mm^3^) admitted to the ICU at the Royal Marsden hospital (London, UK) between October 2010 and December 2012 with a diagnosis of severe sepsis/septic shock were included retrospectively. Data were collated from patients' electronic records after approval by the hospital audit committee. Data are presented as the absolute value (%) or median (IQR). Fisher's exact test or the Mann-Whitney U test was used as appropriate.

## Results

Sixty-four neutropenic patients were admitted to the ICU during this period. Forty-seven (73%) patients survived to ICU discharge and 34 (53%) patients to hospital discharge. Twenty-two (34%) patients were alive at 6 months and 18 (28%) patients were alive at 12 months. Seventy-seven percent of patients had microbiologically documented infections. There was no significant difference between ICU survivors and nonsurvivors in duration of neutropenia, the presence of, or removal of, indwelling catheters, or the source of sepsis. Mechanical ventilation and need for vasopressors were associated with worse outcomes. Patient characteristics are presented in Tables 1 and 2.

**Table 1:** Patient characteristics

	ICU survivors	ICU nonsurvivors
*n*	47(73%)	17(27%)
Age (years)	53 (38 to 65)	49 (33 to 62)
BM transplant	24 (51%)	7 (41%)

**Table 2:** Characteristics on admission to the ICU

	ICU survivors	ICU nonsurvivors
Neutrophil count	0.5 (0.3)	0.2 (0.3)^*^
Days of neutropenia	3 (0 to 8)	6 (0 to 19)

*P *< 0.05

## Conclusion

In a group of oncology patients admitted to the ICU with neutropenia and severe sepsis/septic shock, we found an in-hospital mortality of less than 50%. This is similar to the general population.
